# Loss of fragile histidine triad (Fhit) protein expression alters the translation of cancer-associated mRNAs

**DOI:** 10.1186/s13104-018-3278-9

**Published:** 2018-03-14

**Authors:** Daniel L. Kiss, William D. Baez, Kay Huebner, Ralf Bundschuh, Daniel R. Schoenberg

**Affiliations:** 1 Center for RNA Biology, The Ohio State University, 484 West 12th Ave., Columbus, OH 43210 USA; 2 Department of Biological Chemistry and Pharmacology, The Ohio State University, 1060 Carmack Rd., Columbus, OH 43210 USA; 3 Department of Physics, The Ohio State University, 191 West Woodruff Ave., Columbus, OH 43210 USA; 4 Department of Cancer Biology and Genetics, The Ohio State University, 460 West 12 Ave., Columbus, OH 43210 USA; 5 Department of Chemistry and Biochemistry, The Ohio State University, Columbus, OH 43210 USA; 6 Division of Hematology, Department of Internal Medicine, The Ohio State University, Columbus, OH 43210 USA

**Keywords:** Fhit, Translational control, Cancer, mRNA sequencing, Polysome gradients, Tumor suppressor

## Abstract

**Objectives:**

In > 50% of cancers tumor development involves the early loss of Fhit (fragile histidine triad) protein expression, yet the mechanistic pathway(s) by which Fhit mediates its tumor suppressor functions are not fully understood. Earlier attempts to identify a Fhit-deficient gene expression profile relied on total cellular RNA and microarray analysis. The data here used RNA sequencing (RNA-Seq) of Fhit-negative and Fhit-positive cells as proof of principle for the impact of Fhit on specific mRNAs, and to lay the foundation for a study using ribosome profiling to identify mRNAs whose translation is affected by *FHIT* loss.

**Data description:**

RNA-Seq was performed on RNA from lines of Fhit-expressing and Fhit-deficient lung cancer cells. This identified changes in the levels of mRNAs for a number of cell survival and cell cycle progression genes. Polysome profile analysis performed on cytoplasmic extracts from Fhit-negative and Fhit-positive cells showed changes in the sedimentation of select mRNAs consistent with changes in translation efficiency. The impact of differential Fhit expression on the turnover of selected cancer-linked mRNAs was determined by RT-qPCR of cytoplasmic RNA isolated at intervals after treating cells with a transcription inhibitor.

## Objective

The expression of the fragile histidine triad gene, *FHIT*, is lost in over half of human cancers [1]. Because Fhit is a cytoplasmic protein with few interacting protein partners, finding both the mechanism and downstream effectors of Fhit genome protective and tumor suppressor activities have been challenging [1, 2]. Early experiments to characterize the enzymatic function(s) of Fhit showed that Fhit binds and cleaves diadenosine triphosphate (ApppA) and other dinucleoside triphosphates in vitro [3, 4]. The ApppA molecules are very similar to free cap dinucleotides (m7GpppN). All mRNAs receive a 5′ cap consisting of 7-methylguanosine linked to the first transcribed nucleotide through a 5′, 5′ triphosphate linkage. Cap dinucleotides are generated as end-products of the 3′–5′ RNA decay pathway. In normal cells free cap dinucleotides are degraded by scavenger decapping enzymes [5]. Interestingly, Fhit has been shown to bind and hydrolyze cap dinucleotides in vitro [6]. The subsequent discovery that Fhit and its yeast homolog Aph1 exhibit scavenger decapping activity, at least in vitro, suggests a mechanism through which it may act as a tumor suppressor and genome caretaker, and may be key to identifying additional downstream effectors [7]. We tested the hypothesis that Fhit regulates cancer linked mRNAs at the translational level. Our initial work showing that TK1 protein expression is translationally controlled by Fhit is consistent with a scavenger decapping-based regulatory mechanism [8]. The current study used RNA-Seq, polysome profiling and mRNA turnover analysis to identify Fhit-mediated changes in additional cancer-linked mRNAs. This laid the foundation for a subsequent study to identify the transcriptome-wide impact of *FHIT* loss on translation [9]. In the latter study ribosome profiling and an independent set of sample-matched RNA-Seq data were used to identify Fhit-mediated changes in ribosome occupancy.

## Data description

The data in this submission come from three different experiments. The first experiment (Data files 1 and 2) is an RNA-Seq gene expression study to characterize global Fhit-mediated changes in gene expression in cytoplasmic RNA harvested from H1299 lung cancer cells where Fhit is expressed or silenced. With a padj significance threshold of < 0.05, the RNA-Seq libraries yielded ~ 900 mRNAs with levels of detection differing in cytoplasm by at least 1.5-fold (Data file 2) in Fhit positive vs. Fhit negative H1299 cells.

Next, we assessed the translation of several newly identified Fhit-affected mRNAs using cDNAs remaining from earlier cytoplasmic polysome gradient fractionation experiments [8]. Polysome gradient fractionation separates protein-RNA complexes based on their mass, and can approximate the translational output of an mRNA based on the number of ribosomes loaded onto it. A total of ten mRNAs, two controls (Rplp0 and PolR2A), and eight cancer-linked mRNAs (ATF6, GDF15, CENPH, CSK1B, SPC25, AURKA, NDC80, and Top2A) were selected from our RNA-Seq data and assessed for changes in translation. The resulting data are presented in Data files 3, 4, and 5 (Table 1).

**Table 1:** Overview of data files/data sets

Label	Name of data file/data set	File types (file extension)	Data repository and identifier (DOI or accession number)
Data set 1	RNA-Seq FASTQ files generated from RNA sequencing	FASTQ files	[10] https://www.ncbi.nlm.nih.gov/Traces/study/?acc=SRP124332
Data file 2	Changes in mRNA levels upon Fhit rescue as assayed by RNA-Seq	MS Excel file (.xlsx)	[11] http://dx.doi.org/10.6084/m9.figshare.5539123
Data file 3	Polysome gradient analysis of translational changes in response to Fhit loss	Adobe acrobat file (.pdf)	[12] http://dx.doi.org/10.6084/m9.figshare.5540473
Data file 4	Numerical qPCR values and calculations for Data file 3	MS Excel file (.xlsx)	[13] http://dx.doi.org/10.6084/m9.figshare.5877033
Data file 5	Quantifying changes in ribosome occupancy when Fhit expression is lost	Adobe acrobat file (.pdf)	[14] http://dx.doi.org/10.6084/m9.figshare.5540146
Data file 6	Assaying changes in mRNA stability in response to Fhit loss	Adobe acrobat file (.pdf)	[15] http://dx.doi.org/10.6084/m9.figshare.5540476
Data file 7	Numerical qPCR values and calculations for Data file 6	MS Excel file (.xlsx)	[16] http://dx.doi.org/10.6084/m9.figshare.5877027
Data file 8	The methods used for the experiments and data analysis	Adobe acrobat file (.pdf)	[17] http://dx.doi.org/10.6084/m9.figshare.5540482

Finally, we determined if Fhit expression altered the stability of the mRNAs studied. Briefly, cells were treated with 5,6-dichloro-1-β-d-ribofuranosylbenzimidazole (DRB), a selective inhibitor of RNA polymerase II, for 0–10 h. RNA was harvested from cytoplasmic extracts at 2 h intervals after DRB treatment began. Fhit-dependent changes in the stability of targeted mRNAs was assayed using RT-qPCR and are reported in Data files 6 and 7.

A detailed description of the methods used to gather and analyze these data is provided in Data file 6.

## Limitations

The key limitation of this study is the small cross section of mRNAs assayed. Of the ~ 900 mRNAs that changed ≥ 1.5-fold in response to Fhit loss, we assessed 10, two controls and eight mRNAs whose gene products were known to be involved in cancer-linked processes. Since our previous work showed that Fhit affects the translation of TK1 mRNA [8] we assayed these 10 mRNAs for effects on translation and mRNA stability. Ideally, we would have assayed more targets. The second limitation was the behavior of our two control mRNAs in the polysome gradient experiments. Namely, the translation rates of our control mRNAs (RPLP0 and POLR2A) change when Fhit is lost. Although the behavior of an ideal control would not change with respect to Fhit levels, this result is actually predicted by our hypothesis of how Fhit mediates its translational control functions as a function of its scavenger decapping activity.

## Abbreviations

*FHIT*: fragile histidine triad gene
Fhit: the *FHIT* gene protein product
CHX: cycloheximide
DRB: 5,6-dichloro-1-β-d-ribofuranosylbenzimidazole
RT-qPCR: quantitative reverse transcription polymerase chain reaction
mRNP: messenger ribonucleoprotein
